# Application of the Pluripotent Stem Cells and Genomics in Cardiovascular Research—What We Have Learnt and Not Learnt until Now

**DOI:** 10.3390/cells10113112

**Published:** 2021-11-10

**Authors:** Michael Simeon, Seema Dangwal, Agapios Sachinidis, Michael Xavier Doss

**Affiliations:** 1 Doisy College of Health Sciences, Saint Louis University, 1 N Grand Blvd, St. Louis, MO 63103, USA; michael.simeon@slu.edu; 2 Stanford Cardiovascular Institute, School of Medicine, Stanford University, 1701 PageMill Road, Palo Alto, CA 94304, USA; sdangwal@stanford.edu; 3 Working Group Sachinidis, Center for Physiology, Faculty of Medicine and University Hospital Cologne, The University of Cologne, 50931 Cologne, Germany; a.sachinidis@uni-koeln.de; 4 Technology Development Division, BioMarin Pharmaceutical Inc., 105 Digital Drive, Novato, CA 94949, USA

**Keywords:** embryonic stem cells, pluripotent stem cells, genomics, artificial intelligence, cardiovascular research, cell replacement therapy, clinical trials, safety pharmacology, drug discovery, disease modeling

## Abstract

Personalized regenerative medicine and biomedical research have been galvanized and revolutionized by human pluripotent stem cells in combination with recent advances in genomics, artificial intelligence, and genome engineering. More recently, we have witnessed the unprecedented breakthrough life-saving translation of mRNA-based vaccines for COVID-19 to contain the global pandemic and the investment in billions of US dollars in space exploration projects and the blooming space-tourism industry fueled by the latest reusable space vessels. Now, it is time to examine where the translation of pluripotent stem cell research stands currently, which has been touted for more than the last two decades to cure and treat millions of patients with severe debilitating degenerative diseases and tissue injuries. This review attempts to highlight the accomplishments of pluripotent stem cell research together with cutting-edge genomics and genome editing tools and, also, the promises that have still not been transformed into clinical applications, with cardiovascular research as a case example. This review also brings to our attention the scientific and socioeconomic challenges that need to be effectively addressed to see the full potential of pluripotent stem cells at the clinical bedside.

## 1. Introduction

The capacity to proliferate indefinitely, as well as the ability to differentiate into almost all phenotypic cells that constitute a mature organism, make human pluripotent stem cells (hPSCs) an attractive versatile cellular source for cell replacement therapies for many degenerative diseases, such as ischemic heart failure, diabetes, Parkinson’s and Alzheimer’s diseases, and age-related macular degeneration and tissue injuries [1,2].

Three types of hPSCs have been reported so far. Human embryonic stem cells (hESCs), first reported by James Thomson’s group in 1998, are derived from human pre-implantation embryos [3]. Since the derivation of hESC requires the destruction of an embryo, it raises ethical concerns, and also, hESC-based clinical trials have suffered from the concerns of immune rejection after transplantation due to their allogenic origins [2,4]. The second type of hPSCs, called human-induced pluripotent stem cells (hiPSCs), first reported by the Yamanaka and Thomson groups in 2007 [5,6] following the breakthrough discovery in 2016 by the Yamanaka group that enabled the reprogramming of terminally differentiated adult somatic cells directly into a pluripotent state, is derived from the transient expression of the reprogramming factors (various combinations of OCT4, SOX2, KLF4, c-MYC, NANOG, and LIN28) in various somatic cells, such as skin fibroblasts, peripheral blood T-lymphocytes, and keratinocytes from hair follicles [7,8]. The third type of hPSCs is derived by somatic cell nuclear transfer, a strategy that was very popular in 1996 with the creation of the sheep Dolly, by which a nucleus from a differentiated cell is transferred into a denucleated ovum [9,10]. The derivation of this latter type of hPSC remains technically challenging and is rarely used [11]. 

Among these three different types of hPSCs, only hESCs and hiPSCs have been largely explored for regenerative medicine and clinical applications, and these two hPSC types have revolutionized biomedical research and regenerative medicine with their unprecedented potential opportunities for cell replacement therapies for many degenerative diseases and injuries [2] for the last two decades ever since their discovery. In addition, hiPSC-based in vitro disease modeling provides invaluable physiologically relevant model systems for deciphering the genetic and molecular basis of many human diseases and paving the way for accelerated drug discovery, safety pharmacology, and precision- and personalized-regenerative medicine. 

The advances of the hPSC in conjunction with functional genomics technologies that are based on microarrays, next-generation sequencing (NGS), genome-wide association studies (GWAS), and more recently, on clustered regularly interspaced short palindromic repeats (CRISPR)-cas gene-editing technology have contributed to an explosion of knowledge for understanding of the etiology and molecular mechanisms of complex diseases with many causative and associative gene mutations. Moreover, the combination of these technologies allows an understanding of human embryonic development and cell lineage specifications [12,13].

In this review, we highlight the scientific advances made in biomedical research and regenerative medicine by hPSC technology, along with the high-throughput genomic and gene-editing methodologies and, also, what we have not learned or not achieved so far with these combined technologies from their earlier anticipated speculative milestones that the scientific community once were hopeful of achieving, with a specific focus on cardiovascular research. 

## 2. Derivation of hiPSCs for Personalized Precision Medicine 

While iPSC technology has been advancing since Yamanaka’s discovery in 2006, the use of integrative viral vectors as a reprogramming technique and c-Myc as one of the reprogramming factors showed clinical concerns due to insertional mutagenesis and genetic alterations and transgene-derived tumor formation, respectively. The nonintegrative methods such as Sendai Virus, minicircles, recombinant proteins, microRNAs, synthetically modified mRNAs, small molecules, and the episomal plasmid delivery of reprogramming factors without c-Myc are safer alternatives for the generation of iPSC cells [14]. Since these nonintegrative methods can avoid the risk of genomic instability, they reduce the risk for translational error and pose a more relevant cellular source for clinical applications [15]. 

## 3. Differentiation of hPSCs to Clinically Relevant Phenotypic Cells 

While significant strides have been made in hPSC differentiation, there remain challenges in the differentiation processes of hESCs and iPSCs, ultimately limiting the widespread use of stem cell technology in research programs and cell replacement therapies. The current state-of-the-art method to induce lineage differentiation from hPSCs involves controlling the differentiation process via the stepwise sequential addition of growth factors and cytokines (Table 1), which are known to play a role during certain steps of differentiation and ultimately induce the phenotypic characteristics (Table 1).

Although this has enabled the generation of various cell types, including cells with features of neural subpopulations (cholinergic and dopaminergic neurons), cardiac muscle cells, and hepatocytes, these cells resemble fetal tissue more than adult tissue, in most cases [16]. The use of incomplete differentiated progeny from PSC may hold risks associated with tumorigenicity and excessive proliferation. Furthermore, deriving mature, terminally differentiated functional cells from hPSCs remains a tedious and inefficient process across different cell lineages. To continue moving stem cell-based therapies to clinical applications, there is a strong need to improve the differentiation process of PSCs and ensure the efficacy of cells generated from PSCs.

## 4. Functional Genomics of hPSCs as a Powerful Tool in the Study of Cardiomyogenesis

The heart is the first fully functional organ formed during human embryonic development and is comprised of different cell types, including cardiomyocytes, cardiac fibroblasts, endothelial cells, and smooth muscle cells. A better understanding of the molecular and cellular mechanisms of cardiac development is an essential prerequisite in the quest aimed at treating congenital heart diseases and a multitude of juvenile and adult-onset heart diseases due to genetic abnormalities. In the past decades, many of the molecular mechanisms, epigenetic mechanisms, signaling cascades, and master regulators of cardiac development have been elucidated from experimental mouse model systems. Unlike nonhuman animal models, PSCs of both murine and human origin directly offer in vitro access for the phenotypic and transcriptomic characterizations of purified cardiovascular lineages in sufficient quantities and contribute to the explosion of knowledge in the understanding of the molecular processes involved in cardiogenesis [44,45,46]. While, on the one hand, hPSCs have offered unprecedented opportunities to study human disease etiology and cardiac development via in vitro disease modeling and the in vitro recapitulation of cardiogenesis, on the other hand, hPSCs have proven to be a versatile source for cell replacement therapies for a wider spectrum of degenerative diseases and debilitating tissue injuries. This, in turn, necessitated a thorough understanding of cardiac development to obtain clinical-grade cardiac phenotypic cells. Functional genomics of hPSC-derived cardiovascular lineages with Affymetrix microarray and NGS methodologies in combination with siRNA/shRNA/CRISPR-mediated knockdown approaches identified novel mechanisms of cardiac development and transcription factors networks that play a critical role during cardiovascular development that would not be technically feasible with conventional embryology methods.

## 5. In Vitro Cardiac Disease Modeling with hPSCs

Cardiovascular diseases are the leading causes of death globally, with an estimated 17.9 million deaths in 2019, representing 32% of all global deaths according to the World Health Organization’s (WHO’s) latest report and with a prevalence of 49.2% in adults ≥20 years of age in the US, according to the American Heart Association’s 2021 update [47]. Most often, cardiovascular diseases are primarily due to genetic predispositions, with inherited cardiomyopathies and cardiac arrhythmic disorders being the most common [48]. 

The reliable recapitulation of human cardiac diseases for the investigation of their etiology and pathogenicity has often been challenging due to the physiological differences between humans and experimental animals, as well as phenotypic differences between human cardiac cells and heterologous noncardiac cell systems, such as human embryonic kidney (HEK) cells or CHO cells, where mutant genes have been expressed in studies aimed at elucidating the pathogenicity of inherited channelopathies. Although primary cardiac tissues and cardiomyocytes and immortalized cardiac cells have been used as alternatives, they have suffered from the major setbacks of limited availability and the limited proliferative capacity of post-mitotic primary cardiac cells and the limited expression repertoire of relevant cardiac genes in the immortalized cells, respectively [49]. These limitations were overcome by hPSC-based in vitro disease modeling, which has provided an unlimited supply of the relevant phenotypic cells from hPSCs derived either from the respective patients and their family matched controls or from the creation of isogenic cell lines from a control hPSC cell line with the relevant mutations genetically engineered with genome-editing tools such as ZFN, TALENs, and CRIPSPR methodologies. The studies from the last 14 years show hPSC-based disease modeling to be a powerful tool both at the single-cell and at the 3D organoid levels and have significantly advanced our understanding of cardiovascular diseases and COVID-19-mediated cardiovascular complications [49,50,51,52].

### 5.1. hPSC-Based Disease Modeling of Monogenic Cardiomyopathies and Arrhythmic Diseases 

The derivation of hPSC cell lines directly from patients with monogenic inherited cardiac indications, along with family- and gender-matched healthy control subjects and subsequent comparative functional evaluations of these patients and control hPSC-derived cardiomyocytes, are relatively straightforward approaches in the dissection of the molecular and cellular mechanisms underlying monogenic cardiac diseases. Alternatively, control hPSCs can be genetically engineered to introduce the genetic mutation present in the index patient, and the cardiomyocytes derived from these control and isogenic mutant cell lines can be used as the in vitro disease model [53]. Generally, monogenic cardiac diseases show variable expressivity, mainly due to the presence of additional genetic variants that modify the disease’s severity and incomplete penetrance. These hPSC-based disease models from the above two approaches can shed more light on the understanding of the pathogenicity of monogenic cardiac diseases, with a wide spectrum of clinical severity among the ethnically diverse patient population. Additionally, the advances in DNA sequencing methodologies enable pinpointing and cataloging every genetic variant present in the patient population via GWAS and custom genome and epigenome analyses. 

### 5.2. hPSC-Based Disease Modeling of Polygenic Cardiac Diseases with Genetic Complexity

Physiologically more relevant experimental model systems are the major critical component in revealing more insights into the molecular and cellular pathogenesis of both monogenic and complex human diseases. Contrary to monogenic cardiac diseases that are often caused by the dysfunction of a single gene, complex cardiac diseases are influenced by the contribution of a multitude of common genetic variants, each having a small individual additive effect on the phenotype, complicating the study of these complex diseases. 

#### 5.2.1. Translational Genomics and hPSC-Based Disease Modeling 

Large-scale genome-wide association studies (GWAS) have cataloged tens of thousands of sequence variants, such as single-nucleotide polymorphisms (SNPs), insertions, and deletions enriched in disease cases against controls, to determine the effect size of genetic variants statistically in order to identify the risk factors of disease etiology associated with a multitude of congenital heart defects and other disorders in different ethnic populations [54,55,56]. Although these risk variants from GWAS highlight the genomic loci and the genes associated with the disease pathogenesis and progression, the functional validation and interpretation of these variants remain challenging, since the vast majority of the sequence variants are merely statistically associated with disease etiology and have no functional evidence in a biological context [57,58,59]. 

Recent advances in genome sequencing have enabled many large-scale quantitative trait locus (QTL) studies that link phenotypic data (trait measurements) to specific regions of chromosomes to explain the genetic basis of variations in complex traits at the cellular and tissue levels at various biological stages. A QTL is a specific region in the genome where a particular sequence variant correlates with the variation of a quantitative trait in the phenotype or the degree of pathogenicity. Extensive gene expression QTLs (eQTLs) have been systematically performed on most primary human tissues in the last decades to gain more insight into the genetic basis of human complex traits via associations of genotypes with the expression levels of genes. Since these primary intact tissues are comprised of multiple phenotypic cells, the eQTL readout was not straightforward in assessing the cell lineage -specific differential effects of sequence variants or their effects in disease-causal cells. To this end, hPSCs offer highly purified phenotypic cells in sufficient quantities for eQTL studies to enable the elucidation of the cell lineage-specific regulation of gene expression, as demonstrated by the discovery of several novel variants and causative genes involved in lipid metabolism in eQTL studies performed on a cohort of hiPSC hepatocytes [11,60]. High-throughput screening for functional impacts of genetic variants in hPSC cardiomyocyte (hPSC-CM) phenotypes can help in assessing the pathogenicity of variants of uncertain significance (Table 2). 

A recent study with isogenic hiPSC lines engineered to recapitulate NAA15 loss of function and missense variants identified in patients with congenital heart diseases with the use of CRISPR gene editing demonstrated that NAA15 haploinsufficiency perturbed the normal function of undifferentiated hiPSCs and provided molecular mechanisms underlying the pathogenicity. This study also showed how to estimate the pathogenicity of variants of uncertain significance in patient-specific hiPSCs and their differentiated cells [12,75,77].

Whole exome sequencing (WES) is a widely used tool in clinical genomics and has become an attractive approach of variant detection in genetic conditions with suspected genetic etiology stemming from protein-coding DNA in the genome. Targeted sequencing of the suspected exons of protein-coding regions of the genome or WES of the entire exons of the protein-coding regions of the genome in proband-parent trios can be a very effective approach in decoding the disease-causing variants of both familial, as well as sporadic forms of the diseases that are caused by de novo variants. 

#### 5.2.2. hiPSC Research Relevance to COVID-19 

The coronavirus disease (COVID-19) pandemic due to the severe acute respiratory syndrome coronavirus 2 (SARS-CoV-2) in 2020 has caused more than 249 million infections cases and 5 million deaths to date, despite the development of vaccines [78]. Besides respiratory complications, 20–30% of COVID-19 patients experience severe cardiovascular symptoms, namely myocardial injury, arrhythmias, viral myocarditis, acute coronary syndrome, and vascular damage, including thromboembolism [79,80,81,82], which indicated a poor prognosis in COVID-19. A recent study enrolling 100 patients reported that 60% of the subjects had concurrent myocardial inflammation. In contrast, 78% of recovered patients had persistent plasma troponin-I elevation 2 to 3 months post-recovery, warranting bigger cohort studies to carefully evaluate COVID-19 long-term cardiovascular consequences, especially in recovered patients from mild infections [83]. hiPSCs and their derivatives were rapidly recognized as relevant in vitro models to understand the molecular insights of SARS-CoV-2-associated cardiovascular damage and side effects of the antiviral drugs used in various solidary trials led by the WHO worldwide in 2020. hiPSC models provided advantages over conventional in vivo models, because one of the primary receptors for virus entry, angiotensin-converting enzyme-2 (ACE2), was not recognized by SARS-CoV-2 in mice [82]. However, it is unclear whether direct virus-induced myocardial injury or overwhelming systemic cytokine production is the main culprit [51]. COVID-19 critical patient sera-exposed hiPSC-CMs proved the long-term effects of infected sera on cardiac electrical and mechanical dysfunctions. These phenotypes remained irreversible to chronic treatment with IL-1B inhibitor Canakinumab [51,84] despite its beneficial effects seen in mild-to-severe patients in an early clinical trial [85].

COVID-19 autopsies confirmed the in vitro phenotypes observed upon the viral infection of hiPSC-CM monolayer cultures or engineered heart tissues, indicating changes in their morphology and cell functions ranging from sarcomeric disruption and nuclear DNA damage to increased apoptosis, leading to the loss of beats and contractile dysfunction [15,82,86]. A recent study using human cardiac organoids composed of hiPSC-CMs and hiPSC-ECs reported that inflammatory mediators such as INF-γ and IL-1β, combined with dsRNA, caused severe diastolic dysfunction [87], thereby indicating that the cytokine storm might play a key role in cardiac damage during COVID-19. However, using more complex organoid systems incorporating immune cell circulation in 3D organoids or vascularized engineered heart tissues would help in-depth investigations of inflammatory responses in the heart and cellular cross-talks [51]. The utilization of hiPSC-CMs and derived organoids to screen new or repurposed drugs for COVID-19 treatment and evaluate their efficacy was evident in recent reports [88,89,90,91]; however, many of these were conventionally used drugs showing harmful cardiotoxic effects [92,93]. For instance, antibacterial drug azithromycin, immunosuppressant azathioprine, anthelmintic drug ivermectin, antimalarial drug chloroquine, and hydroxy-chloroquine [94,95,96] were used particularly during the early phase of the COVID-19 pandemic in many countries. BRD inhibitor INCB054329 was identified from an inhibitor compound library screen using human cardiac organoids and was demonstrated to prevent diastolic cardiac dysfunction by inhibiting epigenetic regulator BRD4, a therapeutic target of COVID-19-associated cytokine storms [87]. Similarly, another study by Gracia et al. screened for the antiviral effects of different protein kinase inhibitors being used in the current clinical trials [97], where berzosertib, an ATR kinase inhibitor involved in the DNA damage response, demonstrated a potent antiviral activity. Future studies using hPSC cardiovascular cells and organoids will promote it as a standard tool for future preclinical safety and toxicity studies for drug repurposing or novel drug candidate screening [51]. 

Besides drug testing, hiPSC-derived cardiomyocytes can be used to experimentally validate risk predictions due to genetic variants, for example, single-nucleotide polymorphisms in *ACE2* and *TMPRSS2* genes encoding for SARS-CoV entry receptors and could predict the COVID-19 severity variations observed in patients [98,99]. Moreover, Ellinghaus et al. reported a gene cluster on the genomic region 3p21.31 in SARS-CoV-infected patients responsible for increased susceptibility to COVID-19 in a genome-wide association study (GWAS) [100]. This cluster contains genes, e.g., SIT1, a sodium transporter and a chemokine receptor, which are more likely to be associated with COVID-19 disease progression. 

### 5.3. Current Unmet Challenges with hPSC-Based Disease Modeling 

Genetic testing and screening for inherited cardiovascular diseases are nowadays cost-effective thanks to the advances in NGS methodologies and novel bioinformatic algorithm development coupled with artificial intelligence with genomic big data. Although genetic testing offers an opportunity for the identification of causative and associative genetic variants and the prognostic and therapeutic values for the patient, it also catalogs hundreds of nonsynonymous coding variants in an individual, adding more complexity in distinguishing the pathogenic from benign variants that classify variants of uncertain significance (VUS). Again, this is further complicated by the clinical heterogeneity in patients where variable disease phenotypes are observed among the same mutation carriers [49]. 

Although the electrophysiological abnormalities of hiPSC-CMs derived from the hiPSC lines carrying a novel VUS in KCNH2 present in an LQTS patient improved upon the correction of VUS in the hiPSC cell line and enabled the classification and validation of this VUS as “pathogenic”, and there have been similar studies with many VUS, including the HCM-associated VUS in MYL3 and LQT7-associated VUS in KCNJ2, where these VUS have been validated as “pathogenic”, it is practically impossible and time-consuming to generate hiPSCs from all patients with VUSs, correct the variant, and functionally validate their pathogenic potential [49,101,102,103,104,105]. To this end, a systematic high-throughput screening of VUS in every causative and associative cardiac disease-linked gene will accelerate the discovery of pathogenic variants in a cardiac disease context. 

One of the major critical requirements for the validation of the VUS in a cardiac disease-linked gene and to better understand the etiology and pathogenicity of a genetic variant is the availability of the relevant phenotypic cells—in particular, chamber-specific cardiac cells and the ventricular, atrial, and nodal phenotypic cells in pure cell populations. The current directed differentiation protocols so far have reported yield heterogeneous populations of cells. hPSC-CMs at the start of differentiation protocols are of an atrial phenotype that later transitions to a ventricular phenotype over a period of culture conditions and, also, is cell line-dependent. Additionally, these hPSC-CMs exhibit fetal cardiomyocyte characteristics in most parts, and they are very immature cells [106,107]. These pose challenges in the study of adult-onset cardiac diseases such as Brugada Syndrome and Early Repolarization Syndromes, where these phenotypes are exhibited by the cardiac cells solely with adult cardiomyocytes characteristics. Obtaining mature, chamber-specific cardiomyocytes is very challenging as of now and needs substantial effort to achieve these phenotypic mature cells in a pure population. 

## 6. Drug Discovery

Since any phenotypic cells of clinical significance, such as cardiomyocytes, hepatocytes, and neuronal cells, can be derived from any individual individuals in billions, and hPSCs can be engineered to contain the genetic variants to mirror the patient’s genotype and phenotype or can be corrected to replace the genetic defect with genome-editing tools, hPSCs have gained a lot of popularity as attractive and more reliable in vitro human models of diseases for accelerated drug discovery and personalized precision medicine. Additionally, hPSCs hold greater potential in performing unbiased high-throughput compound screening with sophisticated high-content image analysis platforms. In addition, microelectromechanical system-based heart-on-chip technology with hiPSC-CM facilitates the development of microdevices recapitulating cardiac function as a very sensitive bioassay platform for accelerated drug discovery and cardiac toxicity studies [108,109]. 

## 7. Safety Pharmacology

Drug-induced cardiotoxicity is a major clinical concern, with almost 2000 drugs in the market being labeled with warnings for adverse cardiovascular effects [110]. Approximately 30% of potential therapeutic candidates were abandoned during their clinical trials from 2011 to 2012 due to concerns of adverse cardiovascular complications [111]. Cisapride, a drug used to treat heartburn and digestive disorders in adults and children, was reported to have caused 300 deaths and 16,000 injuries due to drug-induced serious ventricular arrhythmias and sudden deaths before its market withdrawal [112,113]. Even with the adoption of US Food and Drug Administration (FDA) recommendations of screening new drugs with the human ether-a-go-go-related gene (hERG) inhibition assay, many market-approved therapeutics such as clobutinolo, sibutramine, tegaserod, rofecoxib, and terfenadine have been withdrawn due to unpredicted drug-induced cardiotoxicity [111,113]. The accurate prediction of the cardiotoxicity of new therapeutic drugs remains one of the major challenges in delivering safer drugs to patients in need. Patient-specific hiPSC-based safety pharmacology screening will enable the identification of adverse cardiac complications at the cardiomyocyte level and also identify high-risk patient populations that are more susceptible to cardiac toxicity induced by the drugs of interest, such as chemotherapy drugs, in case there are no alternatives available. This paves the way for personalized medicine for each and every patient. Additionally, it will help to cut the high costs of drug development and increase the likelihood of discovering novel drugs with no or minimal adverse side effects to the patients [111,114]. 

### Artificial Intelligence-Assisted hPSC-Based Safety Pharmacology Platforms 

Artificial intelligence (AI) is the general term used to classify machines that mimic human intelligence, and its subtypes—machine learning and deep learning—have been used for accelerated high-throughput image content analyses in accelerated drug discovery and safety pharmacology screenings. Machine learning is the practice of using algorithms to preprocess data from training datasets and then make a prediction based on the training datasets. Deep learning is a subset of machine learning that is essentially a neural network that can process unstructured data such as images. Machine learning needs a user input hierarchy of labeled features for prediction model building, whereas deep learning can auto-extract features that can be used as training datasets for prediction model building. Novel high-throughput, high-content images, and automated platforms that utilize human iPSC-derived 3D-engineered cardiac tissue constructs to better recapitulate heart functions and drug responses are being developed and are becoming sophisticated, with comprehensive profiling of the cellular responses to drugs across multidimensional parameter spaces. Artificial intelligence’s machine learning and deep learning approaches have been shown to handle multidimensional datasets in an automated fashion to accurately predict the contractile behavior of hPSC-CMs exposed to cardioactive drugs and have proven to be very powerful tools for more reliable predictions of cardioactive drug-mediated cellular responses [113,115]. 

## 8. hPSC-Based Cell Replacement Therapy and Clinical Trials

Since hiPSCs can be virtually derived from any patient and can be expanded and differentiated to obtain clinical-grade cardiomyocytes in billions, hPSC presents an unprecedented opportunity for cell replacement therapy due to heart failure. In vivo preclinical studies conducted in small and large animals to evaluate the efficiency and safety of hiPSC-CMs have demonstrated that hPSC-CMs can form human cardiomyocyte grafts upon cellular transplantation, can beat in synchrony with the host syncytium, and can improve heart functions in injured hearts for up to 12 weeks [116,117,118,119,120,121]. An in vivo study with a co-injection of hiPSC-CMs and human mesenchymal stem cells in acutely an infarcted swine heart model and a study with the placement of a sheet of hiPSC-CMs over the infarcted region in an ischemic swine model reported improved cardiac performance, angiogenesis, and reduced left ventricular remodeling 8 weeks post-implantation [122,123]. A study with nonhuman primate models using Macaques showed that transplanted hESC-derived cardiomyocytes engrafted with extensive remuscularization occurred at the infarcted cardiac site. At the same time, nonfatal ventricular arrhythmia occurred in all of these animals, and this observation highlights the potential arrhythmic complications that need to be overcome for the safe clinical use of hPSC-CMs [124]. The currently ongoing four clinical trials with (1) implanting cell sheets comprised of allogeneic hiPSC-CMs on the epicardium of patients with heart disease in Japan: Japan Registry of Clinical Trials (JRCT) Trial ID: jRCT2053190081, (2) hiPSC-derived myocardium in Germany (ClinicalTrials.gov accessed on 6 November 2021, Identifier: NCT04396899), (3) endocardial injection of hiPSC-CMs to treat congestive heart failure (ClinicalTrials.gov accessed on 6 November 2021, Identifier: NCT04982081), and (4) hiPSC-derived cardio-spheroids to treat patients with heart failure (ClinicalTrials.gov accessed on 6 November 2021, Identifier: NCT04945018) are demonstrating the potential clinical applicability of hiPSC-CMs to near fruition, as these cells have been touted as a novel versatile cellular source for cell replacement therapy in the last decade. To this end, there are primarily three major challenges to see the true realistic potential of hiPSC-CM-based cell replacement therapies—tumorigenicity, heterogeneity, and immunogenicity.

### 8.1. Tumorigenicity

While hPSC’s infinite proliferation potential poses advantages in obtaining billions of clinically relevant phenotypic cells for cell replacement therapy, this property is a double-edged weapon; if the transplanted cells keep proliferating, they will cause tumors. Three possible scenarios can result in teratoma formation: (1) The hPSCs may contain residual reprogramming factors that may keep the cells dividing even after differentiation into the desired phenotypic cells or in their progenitor stage. (2) Contamination of the final cell replacement product with one or a few undifferentiated hPSCs can result in a tumor over a period of time. (3) hPSCs and their derivatives can accumulate chromosomal abnormalities or undergo genome instability and may acquire a tumorigenic phenotype [2]. To overcome the above tumorigenic possibilities, more efficient directed differentiation protocols that yield high cardiomyocytes and more stringent purification procedures to eliminate undifferentiated hPSCs and to meet with the highest safety standard set for clinical trials and as additional safeguards are critically needed. To eliminate the transplanted hPSC-CMs in the extreme possible scenario of a tumorigenic outcome with the transplanted cells, suicidal cell strategies such as mismatched HLA alleles between the recipients and transplanted hPSCs need to be developed as a preemptive contingency plan. The discontinuation of immunosuppressants in the case of transplanted cells with mismatched HLA alleles will eliminate the transplanted cells if they become tumorigenic [125]. To eliminate the tumorigenic potential due to residual reprogramming factor expression in hPSCs, reprogramming methods with the use of nonintegrating nonviral methods such as mRNA-based reprogramming protocols can be used. To rule out any tumorigenic scenario in hPSCs due to genetic abnormalities, whole genome sequencing (WGS) with improved bioinformatic algorithms to detect cancer-driving mutations with extremely low allelic frequencies are needed.

### 8.2. Heterogeneity

Heterogeneity is an important issue with hPSC-CMs mentioned earlier in hPSC-based disease modeling. Directed differentiation protocols yielding high-purity chamber-specific cardiomyocytes with appropriate naturalistic characteristics need to be developed to reap the full clinical potential of hPSC-CMs for cell therapy after heart failure [126,127].

### 8.3. Immunogenicity

Immune rejection is another major critical issue in hPSC-based cell therapy. There have been controversial reports on the immunogenicity of autologous iPSCs, and more recently, de nova mutations in mitochondria have been implicated as a potential source of neoepitopes of autologous iPSCs [128]. One can use immunosuppressants to overcome the immunogenicity of autologous iPSCs, in case the transplanted cells elicit an immune response. Alternatively, the recently reported HLA cloaking approach where all the HLA genes can be inactivated in hPSCs by deleting their common component beta-2 microglobulin (B2M) and transactivators essential for the transcription of class II MHC genes with CRISPR technology. One caveat with this HLA cloaking approach is that the cells that lack class I MHC will be lysed by natural killer cells; in which case, the activation of inhibitory receptors on NK cells can suppress the cellular lysis of the hPSC cells without HLA by NK cells.

## 9. The Stumbling Blocks in the Translation of the hPSC Cardiomyocytes to Clinical Settings

The clinical potential of hPSC-CMs are challenged by the hPSCs’ intrinsic problem of the tumorigenicity of undifferentiated hPSCs in the final hPSC-CM product intended for transplantation, tumorigenicity, and teratoma formation by the genetic abnormalities in hPSC-CMs; the heterogeneity of the cardiac subpopulation, such as atrial, nodal, and ventricular phenotypes existing in the same hPSC-CMs that might create an arrhythmogenic substrate upon transplantation and immaturity problems of hPSC-CMs, where these cells resemble fetal cardiomyocytes rather than adult cardiomyocytes. Although these challenges can be overcome by the continued research in this regenerative medicine arena, the support for stem cell research has been substantially slimmed down worldwide. Apparently, although governments and healthcare institutions continuously support and recognize the importance of the stem cell-based regenerative medicine field, hPSC-based therapeutic applications in clinical settings are still missing. Moreover, ethically, this research field should have the highest priority in comparison to other scientific challenges supported by governments and, in the meanwhile, by private entities. While there is enormous support for extraterrestrial space explorations and space tourism ventures by private entities, there is not enough support from private entities to unleash the true potential of hPSC-based regenerative medicine to treat millions of patients worldwide suffering from degenerative diseases. This is mainly due to the fact that investors are looking for short-term gain, and they are not willing to support high-risk, high-reward projects with hPSCs. Thus, the socioeconomic constraints on hPSC research support, with adequate funding delays, the tremendous hope that hPSCs once imparted on us in treating millions of people with degenerative diseases into reality.

## 10. Conclusions and Future Perspectives

hPSC holds greater potential as a versatile cellular source for cell replacement therapy in heart failure, accelerated drug discovery, safety pharmacology, and many more applications. hPSCs have greatly advanced our understanding of human cardiac development and the molecular mechanisms of inherited cardiac diseases. hPSCs associated with genome editing methodologies and next-generation sequencing (NGS) methodologies have greatly advanced biomedical research via the creation of isogenic hPSC cell lines as a control for hPSCs with disease-specific mutations and, also, in creating a multitude of hPSC lines with gene mutations for the in vitro modeling of human diseases with complex genotypes and phenotypes. hPSC research and clinical trials need sufficient resources at a level that are being given for space exploration and extraterrestrial life explorations to unleash the full potential of hPSC-based cell therapies to cure and treat human diseases with more vigor in a shorter period of time. There is no doubt that hPSC-based therapies will soon be available to treat patients globally, but this goal can be only be reached in the near future if we are prepared to make huge global investigations into this field.

## Figures and Tables

**Table 1:** List of clinically relevant phenotypic cells derived from hPSCs by differentiation protocols and their purity at the end of the differentiation protocols.

Organ System	Cell Type	Purity Achieved	Associated Pathologies	References
Brain	NPC/astrocytes	N/A	Stroke, Alzheimer’s SCI	[16,17,18]
	b. Oligodendrocytes Progenitor cell	80–90%	multiple sclerosis, spinal cord injury	[18,19]
	c. Microglia	>97.2%	neurodegeneration, neuroinflammation, traumatic brain injury	[19,20,21]
Lungs	alveolar type II cells	N/A	Idiopathic pulmonary fibrosis, SARS-CoV-2	[22,23]
	b. multiciliated airway epithelial cell	N/A	Asthma, PCD	[24]
	c. endothelial cells	75%	familial pulmonary arterial hypertension	[25]
Heart	cardiomyocytes	>99% 78%	LEOPARD syndrome Hypertrophic cardiomyopathy	[26] [27]
	b. cardiac endothelial cells	NA	congenital valve abnormalities	[28]
Liver	End-stage hepatocytes	nearly 100%	non-alcoholic steatohepatitis and fatty liver disease	[29]
	b. multicellular liver organoid	N/A	primary liver cancer, acute liver failure	[30]
	c. liver buds	N/A	acute liver failure	[31]
Pancreas	insulin-secreting beta-cell	N/A	diabetes,	[32,33,34]
Gut	intestinal epithelium enterocytes/hepatocytes		inflammatory bowel disease (IBD),	[35,36]
Kidney	Renal progenitor cells	63.8 ± 3.3%	Kidney disease, acute kidney injury (AKI)	[37]
	b. IM cell (intermediate mesoderm cells)	N/A	chronic kidney disease (CKD), end-stage renal disease (ESRD)	[38,39]
	c. metanephric and mesonephric NPs, ureteric buds	N/A	Kidney disease	[40]
Eye	retinal ganglion cell-like neuron retinal pigment epithelium cell corneal endothelium cell	N/A	Retinopathy, Age-related Macular—degeneration (AMD), glaucoma, corneal edema	[41,42,43]

**Table 2:** List of cardiovascular diseases and their pathogenic variants identified from hiPSC-based disease modeling in combination with genome editing and NGS methodologies.

Disease Name	Causative Gene	Mutation/SNP	Altered Signaling Pathway	Technology Used	References
Barth Syndrome	TAZ gene	point mutations, deletions, and duplications	Mitochondrial ROS production and energy metabolism.	CRISPR/Cas9	[61,62]
Type I Brugada Syndrome	SCN5A gene	Single-Nucleotide Polymorphism	Inward sodium current pathway, increased triggered activity, and abnormal calcium transients	CRISPR/Cas9	[63]
Long QT syndrome	CALM1-3 gene	relatively unknown	Abnormal electrophysiological properties of LQT15-hiPSC-CMs which was prolonged APD (dominant-negative suppression of LTCC inactivation)	Cas9 double nickase system	[64]
Long QT syndrome	KCNH2 gene	heterozygous c.A2987T mutation	*I*_Kr_ reduction with consequential action potential (AP) duration (APD) prolongation	Homologous recombination using Cre recombinase	[65]
Cardiomyopathy	LMNA gene	frameshift mutation	PDGF signaling pathway	TALEN	[66]
Congenital defect of the bicuspid valve	N1 gene	heterozygous nonsense mutations in *N1*	Notch signaling pathway	TALEN	[28]
Doxorubicin-induced cardiotoxicity (DIC)	*RARG*-S427L gene	missense mutation	Differential regulation pathway of topoisomerase IIβ (*TOP2B*)	CRISPR/Cas9	[67]
Bicuspid Aortic Valve (BAV)	GATA4	missense mutation	The transition of endothelial into mesenchymal cells (EndoMT pathway), a critical step in heart valve formation	CRISPR/Cas9	[68]
Arrhythmogenic Right Ventricular Dysplasia/Cardiomyopathy (ARVD/C)	*SCN5A*	missense desmosomal mutation	Amino acid substitution in Nav1.5 revealed changes in the sodium current amplitude and structural deficit in the organization of a protein directly relevant to cell adhesion (N-Cadherin)	CRISPR/Cas9	[69]
Fabry Cardiomyopathy	GLA gene	Base substitution at intron 4 and insertion between exon 4 and 5	Proinflammatory pathway; NF-κB and MAPK signaling pathway.	CRISPR/Cas9	[70]
Marfan Syndrome	*FBN1*	missense mutation at exon 30	Inhibition of fibrillin-1 and TGF-β pathway	CRISPR/Cas9	[71]
Dilated cardiomyopathy	RBM20	missense mutation	Impaired interactions with spliceosomal proteins	CRISPR/Cas9	[72]
Dilated cardiomyopathy	PLN	PLN R14del mutation	Ca(2+) handling abnormalities, electrical instability	TALEN	[73]
Dilated cardiomyopathy	SPEG E1680K	missense mutation	Striated muscle enriched protein kinase pathway	CRISPR/Cas9	[74]
congenital heart diseases	NAA15	loss of function and missense variant	Consequences of amino acid sequence variants of unknown significance on NAA15 function	CRISPER/Cas9	[75]
Friedreich’s ataxia	FXN	intronic expansion of GAA repeats	Altered iron homeostasis regulation	ZFN	[76]

## Data Availability

Not applicable.
